# Acute exposure to blue wavelength light during memory consolidation improves verbal memory performance

**DOI:** 10.1371/journal.pone.0184884

**Published:** 2017-09-18

**Authors:** Anna Alkozei, Ryan Smith, Natalie S. Dailey, Sahil Bajaj, William D. S. Killgore

**Affiliations:** Department of Psychiatry, University of Arizona College of Medicine, Tucson, Arizona, United States of America; Waseda University, JAPAN

## Abstract

Acute exposure to light within the blue wavelengths has been shown to enhance alertness and vigilance, and lead to improved speed on reaction time tasks, possibly due to activation of the noradrenergic system. It remains unclear, however, whether the effects of blue light extend beyond simple alertness processes to also enhance other aspects of cognition, such as memory performance. The aim of this study was to investigate the effects of a thirty minute pulse of blue light versus placebo (amber light) exposure in healthy normally rested individuals in the morning during verbal memory consolidation (i.e., 1.5 hours after memory acquisition) using an abbreviated version of the California Verbal Learning Test (CVLT-II). At delayed recall, individuals who received blue light (n = 12) during the consolidation period showed significantly better long-delay verbal recall than individuals who received amber light exposure (n = 18), while controlling for the effects of general intelligence, depressive symptoms and habitual wake time. These findings extend previous work demonstrating the effect of blue light on brain activation and alertness to further demonstrate its effectiveness at facilitating better memory consolidation and subsequent retention of verbal material. Although preliminary, these findings point to a potential application of blue wavelength light to optimize memory performance in healthy populations. It remains to be determined whether blue light exposure may also enhance performance in clinical populations with memory deficits.

## Introduction

Short-wavelength light exposure (~480nm, blue light) plays multiple important roles in biopsychological functioning. Specifically, in addition to its role in conscious visual perception through the lateral geniculate nucleus and projection to primary and secondary visual cortex, light exposure can also influence the timing of circadian rhythms, the magnitude of alertness, and quality and duration of sleep through a secondary non-image forming light response system [1, 2]. When light strikes the retina, the blue wavelengths specifically stimulate intrinsically photosensitive retinal ganglion cells (ipRGCs), which respond by transmitting irradiance signals to a number of sub-cortical nuclei, including the the suprachiasmatic nucleus (SCN) and other nuclei of the hypothalamus. The SCN serves as the body’s master clock and regulates the production of melatonin (a hormone secreted by the pineal gland that prepares the brain for sleep) and circadian rhythms of sleep and wake [2]. In addition, the SCN has projections to the locus coeruleus (LC) in the brain stem [3]. Acute short bursts of exposure to blue wavelength light have been shown to increase activation in the brainstem, in an area consistent with the brain coordinates of the LC [4]. Importantly, stimulation of the LC has been shown to promote greater release of norepinephrine throughout the cerebral cortex [5], which in turn influences a variety of brain functions including alertness [6]. It has therefore been proposed that blue light may activate the LC through projections from the SCN, and that such stimulation of the LC may lead to increased norephineprhine release throughout the brain which in turn increases alertness [4].

Blue light exposure (or bright light more generally) at night leads to increases in subjectively and objectively measured alertness and vigilance, likely as a consequence of suppression of melatonin production [7–9]. However, studies have also shown that blue light (or blue-enriched white light) exposure during the day, a time when melatonin levels are naturally low, also leads to an increase in alertness and vigilance, as well as improvements in working memory performance [10–12]. For example, we have recently shown that 30 minutes of exposure to blue versus amber (placebo) wavelength light during the day led to *subsequently* faster performance on a working memory task (i.e., 45 minutes after light exposure) and increased functional brain responses in regions that are important for working memory processes, such as the dorsolateral and ventrolateral prefrontal cortex (DLPFC and VLPFC) [10]. This alerting effect has even been demonstrated in visually blind individuals, further suggesting that it is produced by activation of the non-image forming ipRGCs [13]. The mechanisms underlying this alerting effect remain to be fully elucidated but one potential explanation that has been proposed as a result of these findings involves the potential stimulating effect of blue light on the LC, leading to increased noradrenergic activation within other areas of the brain (i.e., the PFC), resulting in increased alertness and speed of responding [4, 10].

While studies have shown that blue light increases performance in both simple reaction time tasks and in working memory tasks, it is unclear whether other aspects of cognition may also be affected. Long-term memory (LTM), in particular, is a critical aspect of cognition that could potentially be affected above and beyond the simple effects of blue light on alertness. Although the effects of blue light exposure on memory have not been studied, evidence suggests that norepinephrine has a positive effect on memory consolidation (i.e., the period after memory acquisition) [for a review see 14]. In particular, a number of animal studies have shown that increases in norepinephrine (as a result of drug administration) after memory acquisition led to better LTM [14, 15]. Importantly, the timing of norepinephrine administration appears to play a crucial role, but the optimal timing of stimulation to enhance LTM is unclear and may depend specifically on the type of memory studied. However, it appears that noradrenergic influences are particularly prominent during later stage memory consolidation processes. For example, studies have shown that rats who were administered beta blockers 2 hours after memory acquisition showed amnesia 48 hours later, whereas no effect was seen when beta blockers were administered 5 minutes after learning [16, 17].

In summary, daytime exposure to blue light has been shown to activate functional brain responses in brainstem areas consistent with the LC, a region which, when stimulated, has been shown to release norepinephrine throughout the brain. Because increased norepinephrine during memory consolidation is known to improve memory due to neuromodulatory effects on multiple LTM-related brain areas, it follows that blue light exposure may therefore enhance memory performance—a prediction that remains untested to date. To fill this critical gap in knowledge, we therefore tested the hypothesis that daytime exposure to blue wavelength light for 30 minutes during memory consolidation (~1.5 hours after encoding) would lead to better verbal LTM performance when compared to equal exposure to a placebo (amber) light.

## Materials and methods

### Participants

Thirty healthy 18–32 year olds (17 female; mean age = 21.87± 3.74) took part in the study. Participants were all right handed, native English speakers, free from psychiatric, neurological, and substance use disorders, and reported a regular sleep schedule of going to bed between 10pm and 1am and waking between 6am and 9am. Participants self-reported that they obtained, on average, 6 hours and 45 minutes (SD = 49 minutes) of sleep the night preceding the day of testing.

### California Verbal Learning Test, Version II (CVLT-II)

The CVLT-II [18] is an individually administered test of verbal memory and associated cognitive processes. Participants completed the immediate recall, short-delay, and long-delay free recall parts of the CVLT-II. Participants were read a list of words and told they would be asked to repeat as many words as possible. This test-recall procedure was repeated 5 times (*immediate recall*, *trials 1–5*). The list consisted of 15 neutral words evenly divided into the following categories: animals, furniture, vegetables, and modes of transportation. After the 5^th^ trial, participants were read a second list (i.e., distractor list) and asked to repeat only words from the second list. Immediately following recall of the second list, participants were asked to recall only words presented in initial list (*short-delay free recall*). Approximately 1.5 hours after the short-delay free recall subtest, participants were asked to recall as many words from the initial list (*long-delay recall*). Raw scores (i.e., total number of words recalled) as well as standard scores (i.e., raw scores converted to norm-referenced scores) were calculated for each trial.

### Beck Depression Inventory (BDI-II)

The Beck Depression Inventory (BDI-II) [19] is a 21-item self-report questionnaire used to assess depressive symptoms over the preceding 2 weeks. The BDI-II has been shown to have good psychometric properties [19]. Scores of 13 or higher have been shown to discriminate well between clinical and non-clinical populations, therefore only participants who scored lower than 13 on the BDI-II were eligible for this study [20]. BDI-II scores were nevertheless included as a covariate in the analysis, as depressive symptoms have consistently been shown to influence CVLT-II performance [18].

### Two-subtest form of the Wechsler Abbreviated Scale of Intelligence (WASI-FSIQ)

The Full Scale-II Wechsler Abbreviated Scale of Intelligence (WASI-II FSIQ) [21] was used as a measure of intellectual ability or “IQ”. The WASI-II FSQI is one of the most widely used intelligence scales and correlates highly (r = .92) with the Wechsler Adult Intelligence Scale-III (WAIS; Pearson Assessment, Inc., San Antonio, TX) [21]. The instrument yields scores for Full Scale IQ, Verbal IQ, and Performance IQ. The WASI-II FSQI was individually administered by a trained research technician under the supervision of a licensed doctoral level neuropsychologist. WASI-II FSQI scores were used as a covariate in the analysis, as IQ has been shown to influence performance on the CVLT-II [18].

### Light exposure protocol

**Fig 1:**
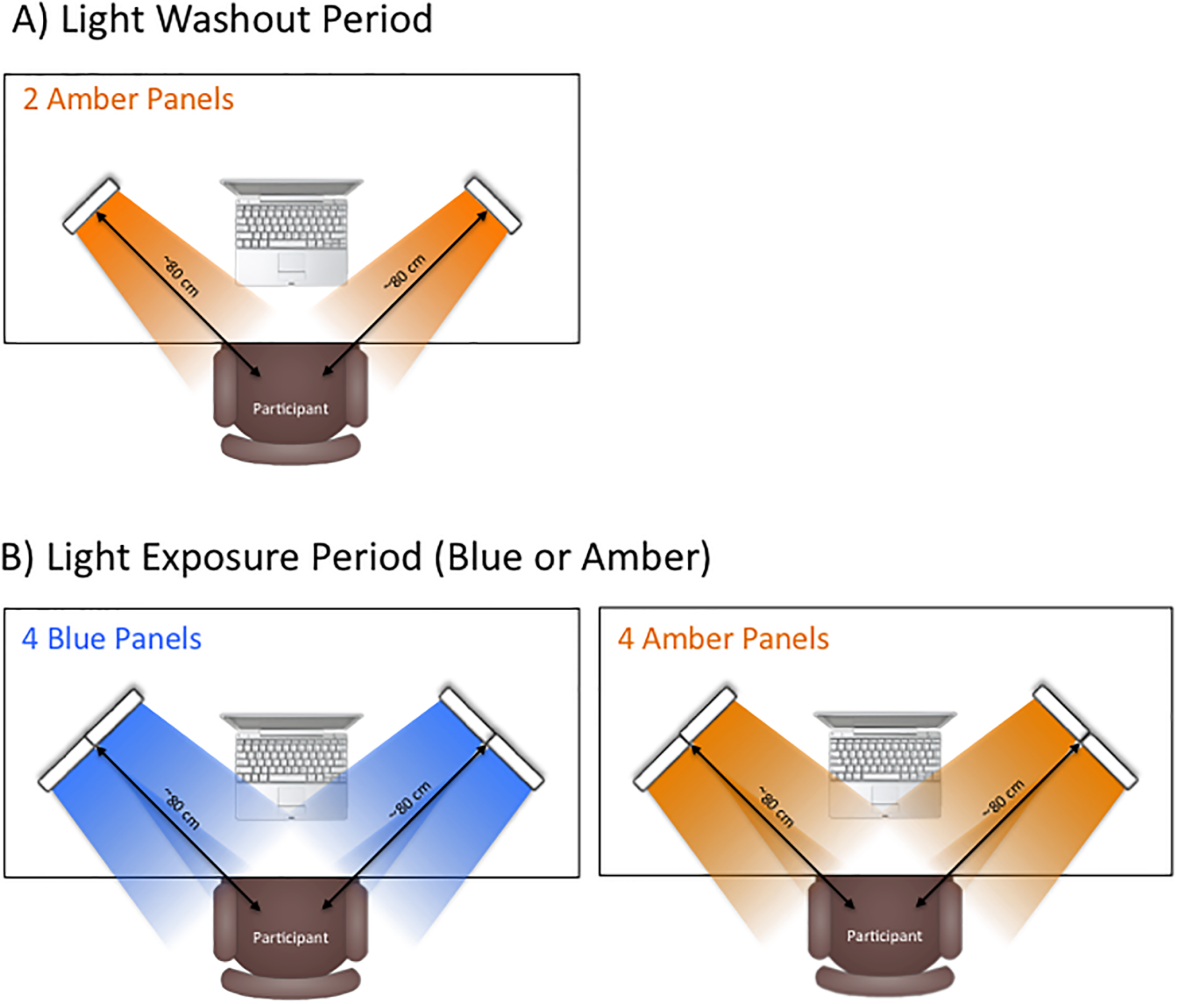
Illustration of the light exposure study design set-up.

The light exposure protocol is described in detail in Alkozei et al. (2016) [10]. In brief, all participants began with a half-hour blue light Washout Period (described in more detail under Procedure) that involved sitting in a dark room while only exposed to two amber light devices (described below) mounted on a desk at a distance of approximately 80 cm from the participant’s nasion, with each light centered at a 45 degree angle from midline (see Fig 1A). During the Exposure Period, light was administered by a similar configuration of four light devices, also centered at 45 degrees to each side of the participant with a distance of approximately 80 cm from the participant’s nasion (see Fig 1B). In the Exposure Period, participants were randomly assigned to undergo a half hour of exposure to an array of either blue or amber light devices. Blue light exposure utilized an array four of commercially available Philips goLITE BLU^®^ Energy Light devices (Model HF3321/60; Philips Electronics, Stamford, CT). Each device consisted of a plastic table-mounted chassis with a 10 x 6 array of light emitting diodes (LEDs), encased in 1 x 1 cm cubical projection elements and a translucent plastic window cover. The goLITE BLU Energy Light is commercially available and has a narrow bandwidth (peaking at λ = 469 nm, at 214 Lux, and single panel irradiance (mW/cm^2^) = 0.11 at 80 cm). The amber placebo devices were provided by the manufacturer for research purposes and were essentially identical to the goLITE BLU devices, with the exception that they were fitted with amber LEDs (peaking at λ = 578 nm, at 188 Lux, and panel irradiance (mW/cm^2^) = 0.04 at 80 cm).

### Procedure

**Fig 2:**
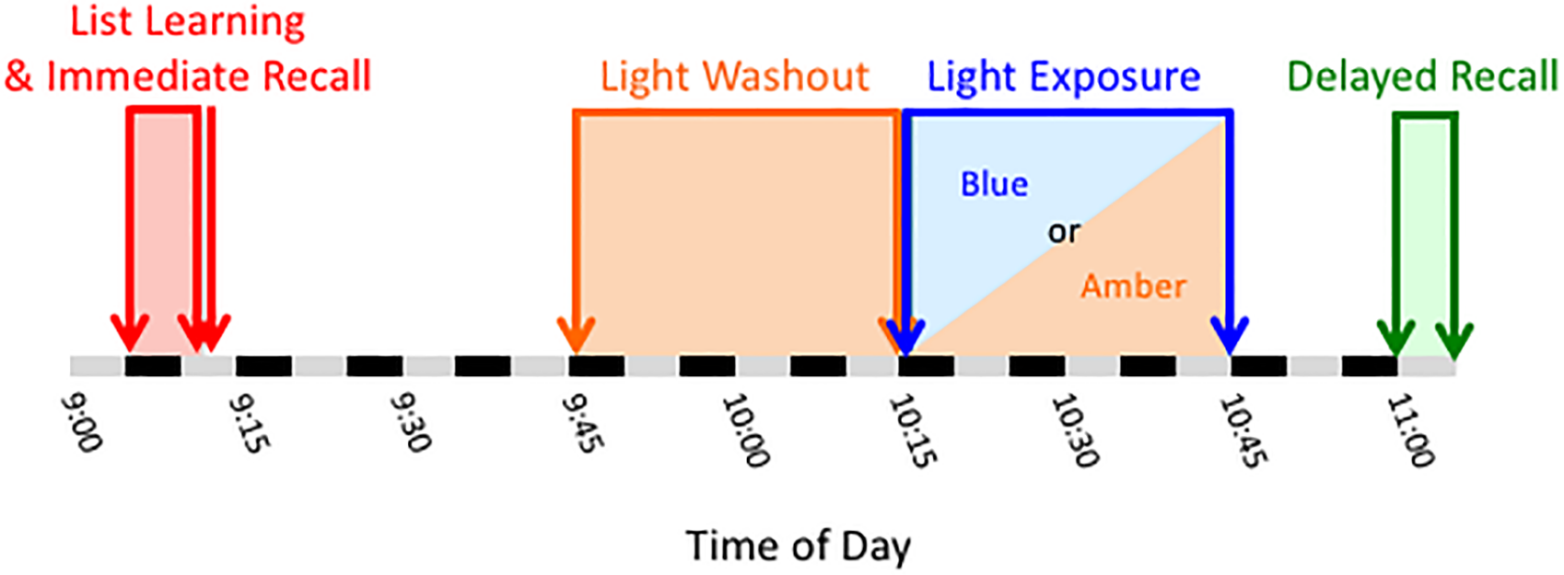
Illustration of the study timeline.

While participants completed the study on an individual basis, all participants were tested at the same time of day to control for circadian time-of-day effects. To avoid potential caffeine withdrawal effects, participants were asked to consume their normal levels of morning caffeine before arrival for the study at 0745. For the first portion of the day, participants completed the informed consent process, basic information questionnaires, and cognitive tasks. At approximately 0905, participants were administered the first 5 encoding trials and the short-delay recall portion of the CVLT-II. Participants were then randomized to receive either 30 minutes of blue (n = 12) or amber (n = 18) light exposure. At approximately 0945, participants underwent the “blue light washout” period (see above) for 30 minutes to ensure that residual effects of outdoor and ambient lighting dissipated before the beginning of the light exposure period. At 1015, the two Washout Period light devices were replaced with the four Exposure Period devices (i.e., either blue or amber). During the two light exposure periods, participants completed a number of computerized tasks. The laptop monitors were fitted with an amber colored Plexiglas panel to block blue wavelength light. At approximately 1100, participants were asked to complete the long delay portion of the CVLT-II. Fig 2 illustrates the timeline of the study design.

## Ethical considerations

The research protocol was reviewed and approved by the Institutional Review Board of the University of Arizona and the U.S. Army Human Research Protections Office. All participants provided written informed consent.

## Data analysis

Change in performance from CVLT-II short-delay free recall to long-delay free recall raw and standard scores between the blue and amber light exposure groups were analyzed using repeated-measures analysis of covariance (ANCOVA), using WASI-FSQI and BDI-II scores as covariates. As the two groups differed in habitual wake time (see Results section below), we also included habitual wake times as an additional covariate.

## Results

### Preliminary analyses

In order to rule out any group differences prior to the light exposure, independent samples t-tests were conducted comparing performance on the CVLT-II between the blue and amber light group. There were no differences in standard scores on the CVLT between the two groups at trial 1 (t(28) = .56, p = .57), trial 5 (t(28) = -1.29, p = .21), or on total performance standard scores (sum of trials 1–5) (t(28) = .17, p = .87). These findings suggest that the two groups did not differ in their initial learning or retention of the word list prior to exposure to the light conditions.

In addition, the two groups did not differ in age, sex, sleep duration the night before the day of testing, number of caffeinated products consumed on the morning before testing, WASI-II FSQI total and Vocabulary subscale scores (see Table 1). Participants also did not differ on habitual bedtime, or habitual sleep duration. However, participants in the amber light group did report significantly earlier habitual wake times (7:20 am; SD = 60 min) than participants in the blue light group (8:07 am; SD = 54 min; t(28) = -2.15, p = .04), and it was therefore included as an additional covariate in the analyses below.

**Table 1:** Descriptive statistics.

	Blue light group n = 12	Amber light group n = 18	Statistic
**Age**	21.50 (3.34)	22.11 (4.07)	t(28) = .42
**Sex**	50% female	61% female	χ^2^(1) = .36
**Sleep duration the night before (in hours)**	6.87 (.71)	6.88 (.90)	t(28) = .05
**Habitual bedtime**	11:33pm (66 min)	11::16pm (55 min)	t(28) = -.70
**Habitual waketime**	8:07am (54 min)	7:20am (60 min)	t(28) = -2.15*
**Habitual sleep duration (in hours)**	7.54 (.78)	7.33 (1.02)	t(28) = -.59
**Number of caffeinated products**	1	3	*χ*^*2*^(1) = .43
**WASI-FSQI**	104.75 (12.82)	106.61 (11.50)	t(28) = .68
**WASI-FSQI Vocabulary Subscale**	54.28 (8.92)	54.75(9.62)	t(28) = -.14
**BDI-II**	1.75 (2.00)	2.94 (3.40)	t(28) = 1.01
**CVLT-II Trial 1 standard score**	0.42 (.86)	.28 (1.25)	t(28) = .57
**CVLT-II Trial 5 standard score**	.45 (.97)	.00 (.91)	t(28) = -1.29
**CVLT Trial 1–5 standard score**	55.83 (8.89)	56.39 (8.75)	t(28) = .16

WASI-FSQI: Wechsler Abbreviated Scale of Intelligence Full Scale-II; BDI-II: Beck Depression Inventory; CVLT-II: California Verbal Learning Test

*p <.05

### Hypothesis testing

The repeated-measures ANCOVA showed a significant main effect of time (F(1, 25) = 5.06, p = .03, d = .09) as well as a group x time interaction (F(1, 25) = 4.39, p = .05, d = .84). Post-hoc pairwise comparisons showed that while there was no significant difference from pre- to post-light exposure for the blue light group (p = .13), there was a significant decline in CVLT standard scores from pre- to post-light exposure for the amber light group (p <.001). However, there was no significant difference between the two groups for CVLT long-delay recall scores (p = .20).

**Fig 3:**
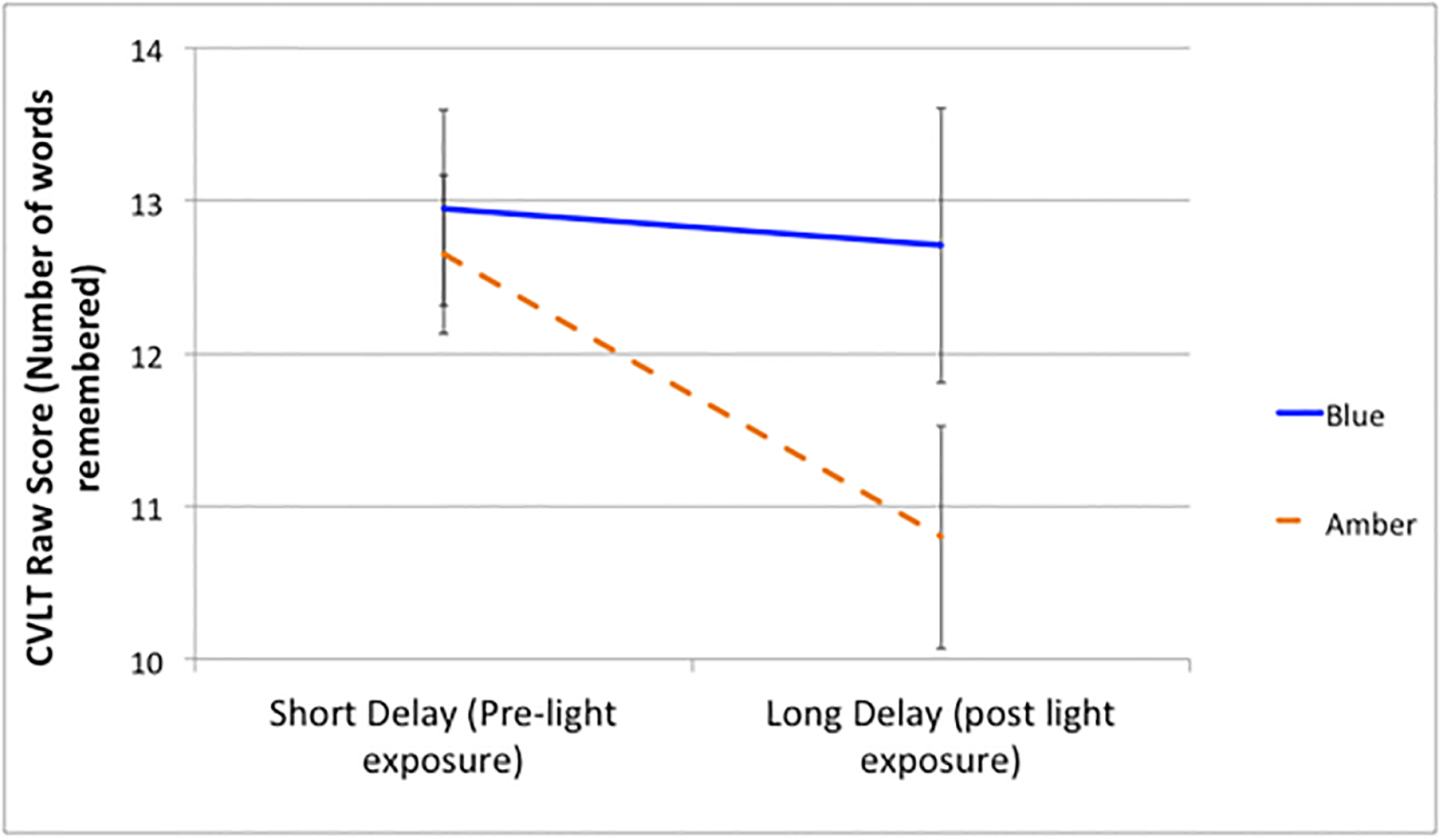
Estimated marginal means and error bars (1SE) for CVLT –II short-delay and long-delay raw scores for individuals in the blue (n = 12) and amber (n = 18) light groups. CVLT-II: California Verbal Learning Test (Version 2).

As standard scores can be difficult to interpret because they include corrections for age and gender, we re-ran the analysis using CVLT raw scores. While, there was no significant main effect of time (F(1, 25) = 2.45, p = .13, d = .62), there was a significant group x time interaction (F(1, 25) = 4.50, p = .04, d = .85). Fig 3 shows participants in the blue light group forgot an average of 0.19 words, whereas participants in the amber light group forgot an average of 1.88 words from short-delay to long-delay recall. This translates to an average decline of only 1.48% in delayed verbal recall for individuals receiving the active blue light treatment, but an average decline of 14.62% for individuals in the amber placebo light group.

## Discussion

The aim of the present study was to investigate the effect of blue wavelength light exposure during memory consolidation on later memory performance in healthy participants. As expected, after learning a list of words, individuals who were exposed to 30 minutes of blue wavelength light during the consolidation period (approximately 1.5 hours after learning) showed greater memory retention than individuals who were exposed to an amber wavelength (placebo) light.

This effect of blue light exposure on memory consolidation was predicted based on its role in promoting activation of the LC [4] which, when stimulated, is known to increase norephinephine release throughout the brain [5]. Norephinephrine, in turn, has been shown to have beneficial effects on LTM systems, leading to enhanced recall [14, 22]. However, while our results provide some initial evidence that blue light exposure sustains better memory recall performance relative to placebo, and the previous literature provides a strong basis for our hypothesis regarding the potential underlying mechanisms, it is important to stress that we did not directly assess neurotransmitter release or brain activation within the noradrenergic system in the present study. It therefore remains necessary for future work to determine whether the beneficial effect of blue light on memory is, in fact, explained by the proposed underlying neural mechanisms.

LTM is the outcome of successful learning and is crucial for normal cognitive functioning. The results from the present study raise the intriguing possibility that blue light exposure during the consolidation period might prove useful as a strategy to optimize the retention of verbal memory. In the present study, we found that those who were exposed to blue light during the consolidation period showed only a 1.48% decline in retention of previously learned words after two hours, compared to a 14.62% decline for those in the placebo group. If confirmed in future work, this strategy could be of potential benefit to nearly any population engaged in active learning, such as school-aged children, college students, vocational students, and those invested in learning even in later adulthood, just to name a few.

While not tested here, it is likely that blue light might also prove beneficial for individuals whose memory is compromised, due to disease or injury. In fact, our results complement a previous longitudinal study (average duration 15 months) that investigated the effects of continuous exposure to either bright light (1000 lux) or dim (300 lux) light in an elderly residential group facility [23]. Older adults who were continually exposed to bright light showed attenuated cognitive deterioration, as measured by the Mini Mental State Examination (MMSE), as well as diminished depressive symptoms when compared to individuals who were exposed to dim light. While the MMSE measures aspects of short-term and long-term memory, it also measures other aspects of cognitive functioning. It is therefore unclear whether bright light influenced learning and memory in particular, or whether other cognitive processes were also positively influenced. In addition, it is unclear how continuous exposure to bright light may influence memory and learning differently when compared to short, targeted exposure to blue wavelength light. Future studies will be necessary to investigate whether the beneficial effects of blue light exposure on memory would also be found using broad spectrum bright light (which contains a large proportion of light within the blue wavelengths), or whether longer durations of bright light exposure would be necessary to achieve the same effect as targeted blue light exposure specifically. However, these results provide promise that using blue (or broad spectrum bright) light may be useful in different settings where learning and memory are important. For example, blue light exposure could be implemented during memory training for elderly individuals, or it could be used selectively by students to improve memory for important test material. In addition, exposure to blue wavelength light from natural sun exposure may have similar beneficial effects on memory; however future research will be necessary to investigate whether the results from this study are also found in such naturalistic settings.

The results from this study focused specifically on the effects of blue wavelength light during memory consolidation (i.e., 1.5 hours after memory acquisition). The present study focused on brief, targeted, blue light exposure within the period where consolidation should be occurring, which resulted in no significant change in word retention and recall after a two hour delay, when compared to an amber placebo group which showed a significant decline in verbal memory performance. It may be that light exposure during memory consolidation is more adventatgeous than light exposure during the learning/encoding phase. However, this is an open question for further research, as it remains unclear whether blue light exposure before, during, or at different time points after learning would lead to similar or enhanced effects. Future studies will be necessary to conduct a systematic comparison of memory performance after blue light exposure at various time points. In addition, this study focused exclusively on verbal memory; thus, the effects of blue light exposure on other types of memory, such as visuo-spatial, temporal, or prospective memory, are unclear and require future investigation. It has also been shown that gray matter volume changes across the menstrual cycle are associated with changes in verbal memory performance [24]. We did not control specifically for menstrual phase in our analyses, so it is conceivable that exposure to blue wavelength light could potentially have different effects for women at different stages of their menstrual cycle. In addition, while 30 minutes of blue wavelength light has been used as a standard duration of exposure across a number of studies [10, 25], it will be necessary to investigate the duration of exposure that is necessary or sufficient to improve memory performance.

One important consideration is the potential role of accumulated sleep debt on testing performance. The participants reported sleeping on average 6.8 hours the night before the assessment, which is slightly less than the recommended 7–8 hours per night for most healthy individuals. Other than self-reported sleep for the night before testing, there was no extensive assessment of pre-study sleep, so it is not possible to establish the extent of sleep debt or whether participants were in fact fully rested when they took part in the study. However, participants were randomly assigned to the treatment conditions, so this should not have influenced the effects of light. It is also worth considering that the study began at 7:40 in the morning, at a time proximal to most participants’ habitual wake up time, suggesting that many individuals may have truncated their sleep time the day of the study. Further, the two groups differed slightly in terms of habitual wake times, with amber normally awakening about 47 minutes earlier on average relative to the blue group. Conceivably, this could have placed the blue group in a particularly suboptimal positioning for cognitive performance relative to the amber group. However, we controlled for this statistically in our analyses and this did not appear to affect the outcomes. As such, it is possible that blue light may in fact lead to enhanced effects particularly when sleep pressure is high. It is therefore unclear whether our findings are generalizable to situations where individuals had the opportunity to be fully rested.

It should also be mentioned that the two light conditions were not equated for light intensity. The light emitted from the blue light devices emitted nearly three times greater irradiance as the light from the amber devices, although they appeared similar in overall visual brightness and averge lux. It is therefore possible that the improvements in memory consolidation seen in the blue light group could be attributed to light intensity rather than light color. However, studies have shown that 50 second bursts of blue light specifically, in comparison to violet light of the same intensity, led to increases in functional brain activation in the LC, supporting our proposed mechanism [4]. Replications of our findings while comparing the effects of different wavelengths of light of the same intensity on memory consolidation are nevertheless needed. In addition, it has been shown that 15 minutes of exposure to orange versus blue wavelength light 1 hour before a second exposure to blue light increased functional brain responses within the prefrontal cortex during a working memory task [26]. It is therefore possible that exposure to amber light during the “washout” period in the present study, led to an enhanced effect of blue light exposure during memory consolidation. This intriguing possibility should be investigated further. Finally, this study was conducted with a relatively small sample of healthy adults; future research will therefore be necessary to replicate these findings across larger sample sizes and perhaps even in clinical populations with memory impairments.

## Conclusion

In summary, exposure to a half hour of blue wavelength light during memory consolidation led to better subsequent delayed verbal memory recall, when compared to an amber (placebo) light condition. These findings may have important implications for clinical populations with memory impairments, as well as for healthy individuals who want to improve their ability to retain newly learned material. Considering this is the first study to investigate whether 30 minutes of blue light exposure can influence memory performance, future research will be necessary to confirm this effect, and to investigate the precise mechanisms, optimal dose/timing of administration, and possible application to clinical samples.

## Supporting information

S1 Dataset Dataset for analyses. (SAV)
